# Toxicity of Hypaconitine from *Aconitum coreanum* (H. Lév.) Rapaics Against the Oriental Armyworm, *Mythimna separata* (Walker)

**DOI:** 10.3390/insects16111080

**Published:** 2025-10-22

**Authors:** Xiuwei Li, Jiaqi Xing, Meng Yang, Naiwei Chen, Yaping Liang

**Affiliations:** Department of Pesticide Science, College of Plant Protection, Shenyang Agricultural University, Shenyang 110866, China; xiuwei001@syau.edu.cn (X.L.); 2023220571@stu.syau.edu.cn (J.X.); yangmengyyy@163.com (M.Y.); cnw0701@126.com (N.C.)

**Keywords:** hypaconitine, insecticidal activity, *Mythimna separata*, growth inhibition, detoxification enzymes

## Abstract

*Mythimna separata* (Walker) (Lepidoptera: Noctuidae) is a major migratory pest causing severe damage to cereal crops such as maize, wheat, and rice across Asia, and is also found in many parts of Oceania. With increasing insecticide resistance, botanical alternatives are urgently needed. This study evaluated the insecticidal potential of hypaconitine, a C19-diterpenoid alkaloid from *Aconitum coreanum*, against *M. separata* larvae. Hypaconitine exhibited significant stomach toxicity and strong antifeedant activity. It also caused pronounced growth inhibition, prolonged larval and pupal development, reduced pupation and adult emergence, induced morphological deformities, and significantly shortened adult longevity. Crucially, biochemical assays revealed sustained, time- and concentration-dependent upregulation of key detoxification enzymes—carboxylesterase (CarE), glutathione S-transferase (GST), and cytochrome P450 (CYP450)—over 72 h, indicating that hypaconitine imposes severe metabolic fitness costs rather than being readily detoxified. These effects collectively demonstrate that hypaconitine’s insecticidal efficacy arises not only from direct toxicity but also from exploiting the physiological trade-offs inherent in xenobiotic defense. Its multi-modal action—combining larvicidal, antifeedant, growth-regulatory, and metabolism-disrupting effects—presents a novel strategy for bioinsecticide development with a lower risk of resistance evolution. These findings highlight hypaconitine as a promising candidate for sustainable, integrated management of *M. separata* and other resistant lepidopteran pests.

## Simple Summary

The oriental armyworm (*Mythimna separata*) is a highly destructive migratory pest threatening cereal crops. With rising insecticide resistance and environmental concerns, novel bio-based alternatives are critically needed. This study evaluates hypaconitine—a diterpenoid alkaloid derived from *Aconitum coreanum*—for its insecticidal potential. We demonstrate that hypaconitine acts not only as a potent stomach poison and antifeedant, but also exploits a key physiological vulnerability in the pest: the high metabolic cost of sustained detoxification. Treated larvae exhibit prolonged development, severe pupal deformities, reduced adult emergence, and shortened lifespan—classic signs of fitness loss. Biochemical analyses confirm strong, prolonged induction of detoxification enzymes (CarE, GST, CYP450), indicating that the insect’s own defense response becomes a liability. By turning the pest’s detoxification system against itself, hypaconitine disrupts multiple life stages with a multi-modal mode of action that reduces the likelihood of rapid resistance evolution. These findings position hypaconitine as a promising, eco-friendly lead compound for next-generation bioinsecticides targeting resistant lepidopteran pests.

## 1. Introduction

The oriental armyworm, *Mythimna separata* (Walker) (Lepidoptera: Noctuidae), is a major migratory pest that inflicts severe damage on cereal crops—including maize, wheat, rice, sorghum, and sugarcane—across Asia and Oceania [1,2]. Due to its long-distance migration and polyphagous feeding behavior, significant crop losses have been documented in China [3], Japan, India, Bangladesh, Pakistan, Australia and New Zealand [4]. Extensive outbreaks of this pest are closely linked to climate change, particularly rising temperatures [4,5]. In recent decades, foliar application of synthetic insecticides has been the primary strategy for managing *M. separata*. However, their indiscriminate use has raised serious environmental concerns and accelerated the development of insecticide resistance [6]. Field populations in China exhibit sensitivity to moderate resistance against chlorpyrifos, lambda-cyhalothrin, chlorfenapyr, and emamectin benzoate, and moderate to high resistance against phoxim [7]. In Pakistan, populations from Punjab province show 109.76- to 148.59-fold resistance to conventional insecticides [8], underscoring the urgent need for alternative, sustainable control approaches.

*Aconitum* species (Ranunculaceae), widely distributed across northern Asia, Europe, and North America, produce highly toxic C19- and C20-diterpenoid alkaloids that are harmful to mammals [9]. Those diterpenoid alkaloids constitute a structurally diverse category of biologically active natural compounds, historically employed as medicines [10,11], poisons [12], and insecticides [13,14]. Aconitine, hypaconitine and related alkaloids found in the *Aconitum* species are highly toxic cardiotoxins and neurotoxins [15]. Severe aconite poisoning can occur after accidental ingestion of the wild plant or consumption of an herbal decoction made from aconite roots [16]. Extracts from plants in the genus *Aconitum* have been used for insect and rodent control. Aconitine demonstrates high contact toxicity against *Plutella xylostella*, *Pieris rapae* and armyworm [17], while alkaloids from *A. sinomontanum* achieved 70.1% antifeedant rate against third-instar *M. separata* larvae [18]. The petroleum ether extract of *A. polycarpum* shows contact toxicity against *Spodoptera exigua* (LC_50_ = 126.2 mg/L), comparable to that of cyhalothrin, and compounds such as L-ascorbyl dipalmitate and octadecadienol exhibit broad-spectrum activity against *S. exigua*, *M. separata*, and *S. frugiperda* [19]. Moreover, an alkaloid from *A. franchetii* var. *villosulum* suppressed carboxylesterase (CarE) activity in *S*. *exigua*, suggesting a potential mode of action involving detoxification enzyme inhibition [20]. Collectively, these findings highlight the potential of *Aconitum*-derived diterpenoid alkaloids as eco-friendly, plant-based insecticides.

Our previous studies confirmed that hypaconitine, isolated from *Aconitum coreanum*, exerts strong stomach toxicity and antifeedant activity against *M. separata* larvae [21,22], and also shows potent larvicidal effects against *S*. *frugiperda* (LC_50_ = 14.99 mg/L) [23]. However, the sublethal impacts of hypaconitine on *M. separata*—including its effects on larval growth, pupation success, adult emergence, morphological development, and key detoxification enzymes (CarE, GST, and P450)—remain uncharacterized. A comprehensive understanding of these multiple physiological and biochemical responses is essential to evaluate hypaconitine’s potential as a resistance-breaking bioinsecticide. However, the sublethal effects of hypaconitine on *M. separata*—including its impacts on larval growth, pupation success, adult emergence, morphological development, and key detoxification enzymes (carboxylesterases [CarE], glutathione S-transferases [GST], and cytochrome P450 monooxygenases [CYP450])—remain uncharacterized. A comprehensive understanding of these physiological and biochemical responses is essential to evaluate hypaconitine’s potential as a resistance-breaking bioinsecticide.

Therefore, this study systematically investigates the lethal and sublethal effects of hypaconitine on *M. separata*, with three specific objectives: (1) to quantify its stomach toxicity and antifeedant activity; (2) to assess its growth-inhibitory and developmental-disrupting effects; and (3) to evaluate its interactions with major detoxification enzyme systems. The findings aim to elucidate the mode of action of hypaconitine and support its development as an eco-friendly tool for managing insecticide-resistant *M. separata* populations.

## 2. Materials and Methods

### 2.1. Insect Rearing and Sources

A susceptible strain of *M. separata* (shenyang, China), kindly provided by Ms. Xiuhui Chang (National Engineering Research Center of Pesticide, Shenyang Sinochem Agrochemicals R&D Co., Ltd.), has been maintained at the College of Plant Protection, Shenyang Agricultural University, China, without exposure to insecticides to preserve its susceptibility. The colony is reared in the pesticide toxicology laboratory under controlled conditions (25 ± 1 °C, 65–75% relative humidity, 14:10 L:D photoperiod, 2000–3000 lx illuminance) and fed fresh corn leaves ad libitum. During routine maintenance, larvae are reared in groups of approximately 200 individuals per rectangular plastic container (30 cm length × 20 cm width × 9.5 cm height). The upper opening of each container is covered with double-layered gauze to ensure ventilation while preventing larval escape.

For the experiments, newly molted second- and third-instar larvae were selected from this colony. Instar identity was confirmed based on head capsule width: 0.50–0.65 mm for second instar and 0.75–1.05 mm for third instar. Within each instar, only individuals within a narrow body size range—second instar: ~5.0–5.5 mm; third instar: ~7.0–7.5 mm—representing early-stage larvae shortly after molting were used to minimize developmental variation. During bioassays, ten larvae were assigned to each 90 mm diameter Petri dish (one dish per replicate) containing treated corn leaves. This group-rearing approach aligns with widely accepted methodologies for antifeedant and stomach toxicity testing in noctuid species, as it balances experimental efficiency with biological relevance under short-term exposure conditions.

### 2.2. Chemicals

Hypaconitine was isolated from the dried tuberous roots of *A. coreanum* (Anguo, Hebei, China) using a conventional acid–base extraction method to yield a crude total alkaloid fraction. The crude extract was first subjected to silica gel column chromatography with gradient elution using petroleum (AR, Aladdin, Shanghai, China) ether–acetone (AR, Xilong, Shenzhen, China) (80:1 to 1:1, *v*/*v*), and fractions were monitored by thin-layer chromatography (TLC) using a commercial hypaconitine reference standard (Xi’an Kai Lai Biological Engineering Co., Ltd., Xi’an, China; purity > 98%) as a guide. Fractions containing hypaconitine were pooled and further purified by a second silica gel column chromatography with chloroform (HPLC grade, Mreda, Beijing, China)–methanol (HPLC grade, Mreda, Beijing, China) (10:1 to 1:10, *v*/*v*) as the eluent, following a previously reported method [24] with minor modifications. The resulting target fractions were concentrated and subjected to preparative high-performance liquid chromatography (prep-HPLC, Hitachi L-2000, Hitachi, Tokyo, Japan) on an Agilent ZORBAX SB-C18 column (250 × 9.4 mm, 5 μm, Agilent, Santa Clara, CA, USA) with a mobile phase of acetonitrile (HPLC grade, Mreda, Beijing, China)–0.1% aqueous formic acid (HPLC grade, Aladdin, Shanghai, China) (25:75, *v*/*v*) at a flow rate of 3.0 mL/min; detection was performed at 235 nm. Elution peaks matching the retention time of the hypaconitine standard were collected, concentrated, and dried to yield a white crystalline solid. The purity of the final compound was determined by analytical HPLC to be ≥98%, and its chemical structure was confirmed by spectroscopic analyses, including infrared (IRAffinity-1S, Shimadzu, Kyoto, Japan) spectroscopy, ultraviolet (UV-2600i, Shimadzu, Kyoto, Japan) spectroscopy, and proton nuclear magnetic resonance (^1^H-NMR, Bruker Avance 600, Bruker, Rheinstetten, Germany), consistent with literature data and the reference standard [21]. The purified hypaconitine was used for all subsequent bioassays.

Triton X-100, sodium dodecyl sulfate (SDS), α-naphthol, and glutathione (GSH) were purchased from Sinopharm Chemical Reagent Co., Ltd. (AR, Shanghai, China). Bicinchoninic acid solution (BCA) and [5,5′-Dithiobis-(2-nitrobenzoic acid)] (DTNB) were purchased from Shanghai Jinsui Bio-Technology Co., Ltd. (BR, Shanghai, China), and 1-chloro2, 4-dinitrobenzene (CDNB) was purchased from Xiya Reagent (AR, Shandong, China).

### 2.3. Larvicidal Bioassay

Second- and third-instar larvae of uniform size and health status were selected for bioassays due to their high sensitivity to xenobiotics and active feeding behavior, which allows clear observation of antifeedant and growth-inhibitory effects. The leaf-dipping method was used to assess the toxicity of hypaconitine against *M. separata* larvae. Based on preliminary range-finding trials, a series of test concentrations (10, 20, 40, 60, and 80 mg/L) were prepared by diluting a stock solution of hypaconitine (100 mg/L) with 0.1% Triton X-100 in anhydrous ethanol. Fresh corn leaves were cut into segments, immersed in the test solutions or the solvent control (0.1% Triton X-100 in anhydrous ethanol) for 10 s, then air-dried on filter paper. The solvent control showed no adverse effects on larval survival in preliminary trials. For the formal bioassay, groups of ten second- or third-instar larvae of uniform size and health status were starved for 6 h and then transferred to 90 mm diameter Petri dishes, each containing five treated leaf segments. Three replicates were performed for each treatment. Petri dishes were maintained in an incubator at 25 °C. Larval mortality and toxicity symptoms were recorded at 48 h and 72 h post-exposure. Viability was assessed by gently prodding the prothoracic tergum and abdominal segments with a blunt needle; a larva was considered dead only if it exhibited no coordinated motor response (e.g., contraction, writhing, or crawling) to two consecutive stimulations applied at a 10 s interval. Mortality data were corrected for control mortality using Abbott’s formula [25]. Dose–mortality relationships were analyzed by probit regression following log-transformation of concentrations and probit transformation of mortality responses. LC_50_ values and corresponding probit regression equations were calculated using DPS software (version 21.5, Hangzhou Ruifeng, Hangzhou, China) [26] based on the corrected data. The Pearson correlation coefficient (R) between log(concentration) and probit(response) was calculated to reflect the strength of the dose–response association in the linearized probit space. Model fit adequacy was independently evaluated using χ^2^ goodness-of-fit tests (*p* > 0.05 for all models).

### 2.4. Determination of the Antifeedant Activity of Hypaconitine on M. separata Larvae

The antifeedant activity of hypaconitine was assessed using the leaf disk method. Third-instar *M. separata* larvae of uniform size and physiological status were selected for the assays. A stock solution of hypaconitine (60 mg/L) was serially diluted with 0.1% Triton X-100 in anhydrous ethanol (AR, Fuyu, Tianjin, China) to yield test concentrations of 5, 10, 15, 20, 25, and 30 mg/L. The solvent control consisted of 0.1% Triton X-100 in anhydrous ethanol, which showed no adverse effects on larval feeding in preliminary trials. Fresh corn leaves were rinsed, and the midribs were removed. Leaf disks (3 cm × 1 cm) were cut and immersed in either hypaconitine solution or solvent control for 10 s, then air-dried on filter paper to allow complete evaporation of ethanol. Ten larvae, starved for 6 h, were placed in each Petri dish (90 mm diameter) containing five treated leaf disks. Three independent biological replicates were performed for each treatment. After 24, 48, and 72 h of feeding, the remaining leaf disks were collected, and the area consumed by larvae was measured using graph paper. Specifically, each leaf disk was placed on millimeter graph paper, and the uneaten area was estimated by counting squares. The antifeedant rate (%) was calculated using the formula:

$$\mathrm{Antifeedant}\;\mathrm{rate}\;(\%)=(\frac{A_{c}-A_{t}}{A_{c}})\times 100$$

where $A_{c}$ is the mean leaf area consumed in the control group and $A_{t}$ is that in the treatment group. Dose–antifeedant response relationships were analyzed by probit regression following log-transformation of concentrations and probit transformation of antifeedant rates. The AFC_50_ (median antifeedant concentration causing 50% feeding inhibition) values and corresponding probit regression equations were calculated using DPS 21.5 software [26]. The Pearson correlation coefficient (R) between log(concentration) and probit (antifeedant rate) was calculated to reflect the strength of the dose–response association in the linearized probit space. Model fit adequacy was independently evaluated using χ^2^ goodness-of-fit tests (*p* > 0.05 for all models).

### 2.5. Analysis of the Growth Inhibitory Activity of Hypaconitine on M. separata Larvae

The leaf-dipping method was adopted to test the growth inhibitory activity of hypaconitine on *M. Separata* larvae. Third-instar larvae of uniform size and physiological status were selected. Preliminary range-finding trials were conducted using hypaconitine concentrations of 0.5, 2, 4, 6, and 8 mg/L. Corn leaf segments were dipped in the respective solutions, air-dried, and then placed in transparent plastic cups (11.5 cm in diameter at the bottom, 15 cm in diameter at the top, and 5.0 cm in height), each covered with a lid fitted with double-layer gauze for ventilation. Ten larvae starved for 6 h were placed in each cup with five dried leaves. Three replicates were conducted per treatment. The larvae fed with 0.1% Triton X-100 anhydrous ethanol-treated leaves served as the control group. After 24 h, 48 h and 72 h of treatment, the larvae were weighed.

### 2.6. Monitoring the Developmental Duration of M. separata Treated with Hypaconitine

The effects of hypaconitine on the developmental duration of *M. separata* larvae were also assessed by the leaf-dipping method. The 3rd instar larvae of uniform size and physiological status were selected for preliminary range-finding trials. The corn leaf segments were treated with 1 mg/L, 2.5 mg/L, 5 mg/L and 10 mg/L hypaconitine. Ten larvae, pre-starved for 6 h, were transferred into transparent plastic cups (11.5 cm bottom diameter, 15 cm top diameter, 5.0 cm height). Each cup was covered with a lid equipped with double-layer gauze (to ensure ventilation) and contained five dried leaves. Three replicates were conducted per treatment. The anhydrous ethanol solution containing 0.1% Triton X-100 was a control group. After 72 h of treatment, the alive tested insects were transferred and fed with artificial feed. The survival condition and the developmental duration of *M. separata* larvae were recorded each day. After pupation, the weight of each pupa was measured, and the key parameters related to growth and development, including pupation rate, average pupal weight, emergence rate were calculated.

### 2.7. Analysis of the Effect of Hypaconitine on the Detoxification Enzymes of M. separata

The larvae alive 24 h, 48 h and 72 h after feeding on hypaconitine-treated leaves were carefully selected, thoroughly washed with water, and dried on filter paper. Each larva was homogenized with 100 μL of 0.1 mol/L phosphate buffer (pH = 7.4, Sangon, Shanghai, China) containing 0.3% Triton X-100 on ice. Then, the homogenate was transferred to a sterilized tube and centrifuged at 4 °C and 15,000 g for 15 min. Finally, the supernatant was collected as the enzyme source and stored at −20° C for subsequent analysis of the detoxification enzymes (CYP450, CarE and GST) and protein assay.

Cytochrome P450 (CYP450) monooxygenase activity was determined by measuring the O-demethylation of *p*-nitroanisole, following the method of Rakotondravelo et al. [27] with slight modifications. Briefly, a 200-μL reaction mixture containing 100 μL of *p*-nitroanisole (0.1 mmol/L in anhydrous ethanol, AR, Macklin, Shanghai, China), 10 μL of NADPH (9.6 mmol/L in 0.1 mol/L potassium phosphate buffer, pH 7.8), and 90 μL of enzyme source was prepared in a 96-well microplate. Reactions without NADPH were run in parallel as blanks to correct for non-enzymatic hydrolysis. The plate was incubated at 30 °C for 30 min in a SpectraMax 190 microplate reader (Molecular Devices, San Jose, CA, USA), and the absorbance of the produced *p*-nitrophenol was measured at 405 nm. Enzyme activity was expressed as nmol of *p*-nitrophenol formed per minute per mg of protein, using an extinction coefficient of ε = 18.3 mM^−1^ cm^−1^. Each sample was analyzed in triplicate (technical replicates), and three independent biological replicates.

Carboxylesterase (CarE) activity was determined using α-naphthyl acetate (98%, Macklin, Shanghai, China) as the substrate, following a standard spectrophotometric method with minor modifications [28]. Briefly, 5 μL of enzyme source was added to a 96-well microplate containing 140 μL of 0.1 mol/L phosphate buffer (pH 7.6) and 40 μL of 0.3 mmol/L α-naphthyl acetate in acetone. The reaction was initiated by the addition of the substrate and incubated at 37 °C for 15 min. The reaction was then terminated, and the released α-naphthol was visualized by adding 15 μL of color developer, prepared by mixing Fast Blue B Salt (1% *w*/*v*, 95%, Macklin, Shanghai, China) and sodium dodecyl sulfate (5% *w*/*v*, AR, Macklin, Shanghai, China) in a 2:5 (*v*/*v*) ratio. After 10 min of color development at room temperature, the absorbance was measured at 600 nm using a SpectraMax 190 microplate reader (Molecular Devices, San Jose, CA, USA). Control wells without enzyme were included to correct for non-enzymatic hydrolysis. CarE activity was expressed as μmol of α-naphthol produced per minute per mg of protein, as determined from a standard curve of α-naphthol (0–100 μM) generated under identical assay conditions. Each sample was analyzed in triplicate (technical replicates), and three independent biological replicates were performed per treatment.

Glutathione *S*-transferase (GST) activity was determined according to the method of Li et al. [29] with slight modifications. The reaction was carried out in a total volume of 220 μL in a 96-well microplate, containing 100 μL of 0.6 mM 1-chloro-2,4-dinitrobenzene (CDNB) [0.0061 g of CDNB was dissolved in 1 mL of absolute ethanol and diluted to 50 mL with 0.05 mol/L Tris-HCl buffer (pH = 7.5), Sangon, Shanghai, China], 100 μL of 6 mmol/L glutathione [0.0922 g of GSH was diluted to 50 mL with 0.05 M Tris-HCl buffer (pH = 7.5)), Sangon, Shanghai, China] and 20 μL of enzyme source. The reaction mixture was pre-equilibrated at 37 °C for 20 min, and the reaction was initiated by the addition of the enzyme source. The increase in absorbance at 340 nm due to the formation of the CDNB–GSH conjugate was monitored every minute for 10 min using a SpectraMax 190 microplate reader (Molecular Devices, San Jose, CA, USA). Control wells without enzyme were used to correct for non-enzymatic conjugation. GST activity was calculated using the extinction coefficient of CDNB–GSH (ε = 9.6 mM^−1^ cm^−1^) and expressed as μmol of product formed per minute per mg of protein. Each sample was analyzed in triplicate (technical replicates), and three independent biological replicates were performed per treatment.

The total protein concentration in each enzyme preparation (used for CYP450, CarE, and GST assays) was determined using the method of Smith et al. [30], with bovine serum albumin (BSA, BR, Sangon, Shanghai, China) as the standard. Briefly, 10 μL of appropriately diluted sample was mixed with 200 μL of BCA working reagent (Beyotime, Shanghai, China) in a 96-well microplate and incubated at room temperature for 30 min. The absorbance was measured at 562 nm using the SpectraMax 190 microplate reader (Molecular Devices, San Jose, CA, USA).

To evaluate the induction effect of hypaconitine on detoxification enzymes, the specific activity of each enzyme (CYP450, CarE, and GST) in treated larvae was normalized to that of the corresponding control group at the same time point (24 h, 48 h, or 72 h). The fold change in enzyme activity was calculated as the ratio of the mean specific activity in the hypaconitine-treated group to that in the control group. Data from three independent biological replicates were used for statistical analysis. One-way analysis of variance (ANOVA) was first performed to determine overall significance, and pairwise comparisons among time points were conducted using the Bonferroni post hoc test to control for Type I error inflation due to multiple comparisons (α = 0.05). All statistical analyses were carried out using DPS software (version 21.5, Hangzhou Ruifeng, Hangzhou, China) [26].

### 2.8. Statistical Analysis

Mortality and antifeedant rates were calculated from raw bioassay data using Microsoft Excel 2021. These values were then used as input for probit analysis, which was performed using the built-in probit regression module in DPS v21.5 [26] to estimate the LC_50_ (lethal concentration causing 50% mortality) and AFC_50_ (median antifeedant concentration causing 50% feeding inhibition), along with their 95% confidence intervals and the slope of the probit regression line. Model goodness-of-fit was evaluated using the Pearson chi-square test, with *p* > 0.05 indicating an adequate fit. For developmental parameters (e.g., larval weight, pupation rate) and detoxification enzyme activities (CYP450, CarE, GST), data normality and homogeneity of variances were assessed using the Shapiro–Wilk test and Levene’s test, respectively, as implemented in DPS v21.5. Percentage data were first arcsine square root transformed to meet parametric assumptions. Although the small sample size (*n* = 3 per group) limits the statistical power of these tests, all datasets yielded *p* > 0.05, supporting the use of parametric ANOVA. Visual inspection of residual plots further confirmed no major violations of model assumptions. Differences among treatment groups were evaluated by one-way analysis of variance (ANOVA), followed by Bonferroni’s post hoc test to control the family-wise error rate at α = 0.05. All data presented are based on three independent biological replicates, with each replicate consisting of larvae collected from a separate rearing batch and processed independently. Technical replicates (e.g., triplicate measurements of the same homogenate in enzyme assays) were averaged prior to statistical analysis to avoid pseudoreplication.

## 3. Results

### 3.1. Insecticidal Activity of Hypaconitine Against M. separata Larvae

The insecticidal activity of hypaconitine against second- and third-instar larvae of *M. separata* was evaluated using the leaf-dip bioassay method. Dose–response assays revealed significant larvicidal effects, with toxicity increasing over time in both instars (Table 1). Second-instar larvae were consistently more susceptible than third-instar larvae, as evidenced by lower LC_50_ values across both 48 h and 72 h exposure periods. This greater sensitivity in younger instars may be attributed to differences in body size, cuticle thickness, metabolic detoxification capacity, or feeding behavior.

Probit regression analyses showed strong dose–response relationships (Pearson correlation coefficients, R = 0.9764–0.9969), and χ^2^ goodness-of-fit tests (all *p* > 0.05) confirmed the adequacy of the models. These results indicate that hypaconitine exhibits potent stomach toxic activity against *M. separata* larvae, with enhanced efficacy in earlier developmental stages and a time-dependent mode of action.

### 3.2. Antifeedant Activity of Hypaconitine Against Third-Instar Larvae of M. separata

Hypaconitine exhibited significant dose- and time-dependent antifeedant effects on third-instar *M. separata* larvae (Table 2). The antifeedant rate increased progressively with both concentration and exposure duration, indicating a cumulative inhibitory effect on larval feeding behavior. Higher concentrations (≥15 mg/L) induced significantly stronger feeding deterrence compared to lower doses, while the response between 15 and 20 mg/L showed minimal difference at 48 h and 72 h, suggesting a saturation effect at intermediate concentrations. Probit analysis confirmed a strong concentration–response relationship at all time points, with all models showing acceptable fit (χ^2^, *p* > 0.05; Table 3). The time-dependent decline in AFC_50_ values further demonstrates that hypaconitine’s antifeedant potency increases with prolonged exposure.

### 3.3. Growth Inhibitory Effect of Hypaconitine on Third-Instar Larvae of M. separata

**Figure 1:**
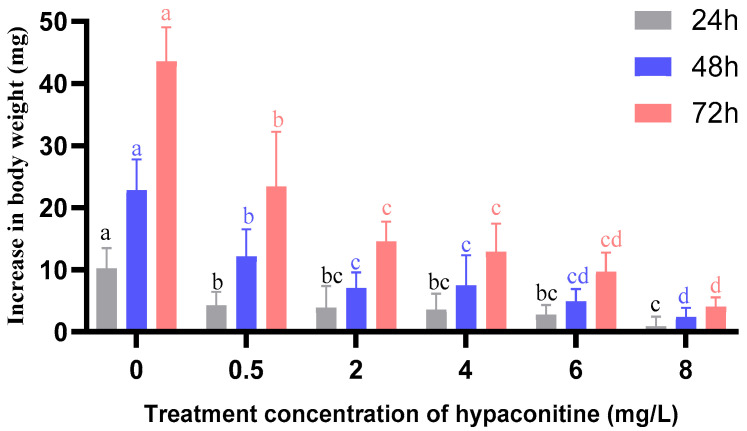
Effect of hypaconitine on the body weight increase of third-instar *Mythimna separata* larvae over time. Note: Values are mean ± standard error (SE) of three replicates. Different lowercase letters above bars indicate significant differences among concentrations at the same time point (*p* < 0.05, Bonferroni’s multiple comparison test). CK represents the control group.

Hypaconitine significantly suppressed the growth of third-instar *M. separata* larvae in a concentration- and time-dependent manner, as reflected by reduced body weight gain over 72 h (Figure 1). All treated groups exhibited dose-responsive growth inhibition relative to the control (CK), with higher concentrations causing progressively greater suppression at every time point. The strongest inhibitory effect was observed at 8 mg/L, where larval weight gain was drastically reduced—remaining markedly lower than that of the control throughout the exposure period. Growth inhibition intensified with prolonged exposure, particularly at elevated concentrations, suggesting a cumulative adverse effect on larval development. Notably, although the degree of suppression increased over time, the relative efficacy of the treatments remained consistent, with the 8 mg/L group consistently exhibiting the most pronounced growth retardation. Statistical analysis confirmed significant differences among all treatment groups at each time point (one-way ANOVA followed by Bonferroni post hoc test, *p* < 0.05), underscoring the potent and dose-dependent growth-inhibitory activity of hypaconitine. These findings highlight its dual potential as both a feeding deterrent and a developmental disruptor in lepidopteran pests.

### 3.4. Effect of Hypaconitine on the Developmental Duration of M. separata

Hypaconitine significantly prolonged the developmental duration of *M. separata* across larval and pupal stages in a concentration-dependent manner (Table 4). Although individual instars showed differential sensitivity—with only the third instar significantly extended at 10 mg/L—the cumulative effect resulted in a significantly longer total larval period at ≥5 mg/L. This delay persisted into post-larval development, as both prepupal and pupal durations were significantly increased at 5 and 10 mg/L. In contrast, adult longevity was significantly shortened at the highest concentration (10 mg/L), suggesting a physiological trade-off between extended immature development and reduced adult lifespan—a pattern consistent with resource allocation constraints under chemical stress.

### 3.5. Hypaconitine Impairs Pupation, Pupal Integrity, and Adult Emergence in M. separata

Hypaconitine significantly impaired key developmental and reproductive fitness parameters in *M. separata*, as reflected by reduced pupation success, elevated pupal deformity, a non-significant reduction in pupal weight, and suppressed adult eclosion (Table 5). Pupation rate was significantly lower than that of the control at all tested concentrations (*p* < 0.05, Bonferroni test), with inhibition intensifying at ≥5 mg/L. Notably, although the numerical difference between 5 mg/L and 10 mg/L was small (56.48% vs. 55.22%), the difference remained statistically significant (*p* = 0.0001), likely due to low within-treatment variability. Pupal deformity increased progressively with hypaconitine concentration, showing significant elevation even at 1 mg/L and reaching 19.45 ± 0.05% at 10 mg/L. Pupal weight exhibited a modest numerical decline across treatments but did not differ significantly from the control (*p* > 0.05). Adult eclosion was markedly suppressed in a concentration-dependent manner, decreasing from 100% in the control to 63.89 ± 0.13% at 10 mg/L. Collectively, these results demonstrate that hypaconitine disrupts multiple stages of post-embryonic development, ultimately compromising pupal viability and adult reproductive potential, and highlight its potential as a biologically active insect growth regulator.

### 3.6. Hypaconitine Induces Time- and Concentration-Dependent Activation of Detoxification Enzymes in Third-Instar M. separata Larvae

**Figure 2:**
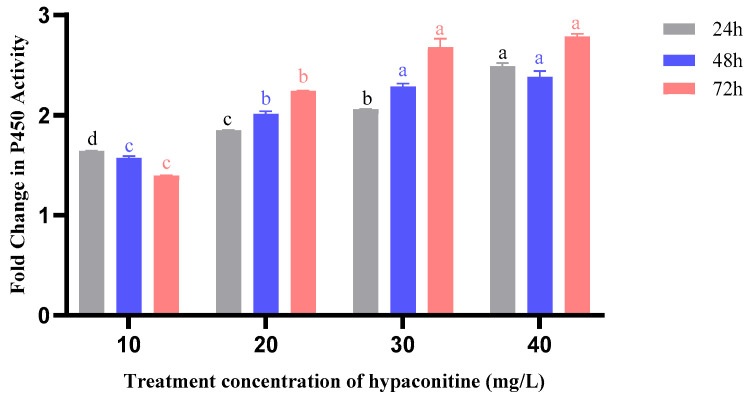
Effects of hypaconitine concentration and exposure time on P450 enzyme activity in third-instar *M. separata* larvae. Note: P450 enzyme activity in third-instar *M. separata* larvae was measured after exposure to different concentrations of hypaconitine for 24 h (gray bars), 48 h (blue bars), and 72 h (red bars). Data are presented as fold change relative to the control and represent mean ± standard error (SE), *n* = 3. Different lowercase letters above bars, color-coded to match exposure duration (24 h: black; 48 h: blue; 72 h: red), indicate significant differences among concentrations within the same exposure time (*p* < 0.05, Bonferroni’s multiple comparison test).

Exposure to hypaconitine triggered a strong, concentration- and time-dependent induction of cytochrome P450 (CYP450) activity in third-instar *M. separata* larvae (Figure 2). At 24 h, enzyme activity increased with rising concentrations, from 1.64-fold (10 mg/L) to 2.49-fold (40 mg/L) relative to the control, indicating an immediate dose-responsive activation. This trend intensified at 48 h (2.38-fold at 40 mg/L) and reached its peak at 72 h (2.79-fold), with all concentrations above 20 mg/L showing sustained induction. The progressive and cumulative activation of CYP450 suggests a central role in the metabolic detoxification of hypaconitine over prolonged exposure.

**Figure 3:**
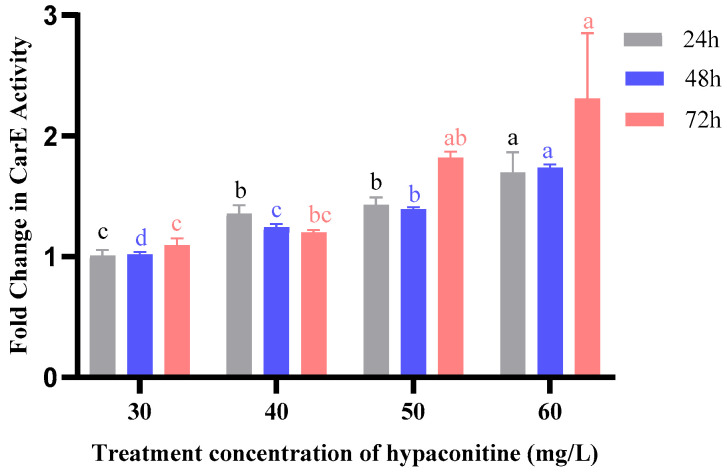
Effects of hypaconitine concentration and exposure Time on CarE enzyme activity in third-instar *M. separata* larvae. Note: CarE enzyme activity in third-instar *M. separata* larvae was measured after exposure to different concentrations of hypaconitine for 24 h (gray bars), 48 h (blue bars), and 72 h (red bars). Data are presented as fold change relative to the control and represent mean ± standard error (SE), *n* = 3. Different lowercase letters above bars, color-coded to match exposure duration (24 h: black; 48 h: blue; 72 h: red), indicate significant differences among concentrations within the same exposure time (*p* < 0.05, Bonferroni’s multiple comparison test).

Carboxylesterase (CarE) activity exhibited a delayed but significant response to hypaconitine treatment (Figure 3). At 24 h, induction was modest, ranging from 1.01-fold (30 mg/L) to 1.70-fold (60 mg/L). By 48 h, activity continued to rise, particularly at higher concentrations. The most pronounced effect was observed at 72 h, where CarE activity reached 2.31-fold at 60 mg/L and 1.82-fold at 50 mg/L, indicating that prolonged exposure is required for maximal enzyme induction. This time-lagged activation suggests that CarE may play a secondary or compensatory role in detoxification, becoming more active as metabolic demand increases.

**Figure 4:**
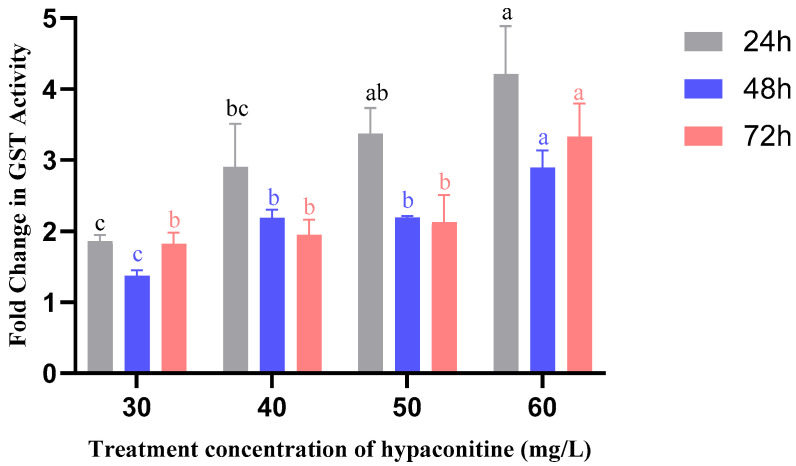
Effects of hypaconitine concentration and exposure time on GST enzyme activity in third-instar *M. separata* larvae. Note: GST enzyme activity in third-instar *M. separata* larvae was measured after exposure to different concentrations of hypaconitine for 24 h (gray bars), 48 h (blue bars), and 72 h (red bars). Data are presented as fold change relative to the control and represent mean ± standard error (SE), *n* = 3. Different lowercase letters above bars, color-coded to match exposure duration (24 h: black; 48 h: blue; 72 h: red), indicate significant differences among concentrations within the same exposure time (*p* < 0.05, Bonferroni’s multiple comparison test).

Glutathione S-transferase (GST) activity showed a rapid and robust induction, peaking at 24 h (Figure 4). At this time point, GST activity increased to 1.86-fold (30 mg/L) and reached a maximum of 4.21-fold at 60 mg/L, the highest fold-change observed among all enzymes and treatments. Although activity declined slightly at 48 h, it remained significantly elevated at 72 h, particularly at 60 mg/L (3.33-fold). This early and intense activation indicates that GST is rapidly recruited in response to oxidative or electrophilic stress induced by hypaconitine, likely involved in conjugation and elimination of reactive intermediates.

Collectively, these results demonstrate that hypaconitine activates multiple detoxification pathways in *M. separata*, with each enzyme exhibiting distinct kinetic and concentration-response profiles. CYP450 shows sustained, dose-dependent induction; CarE responds progressively over time; and GST is rapidly and strongly activated, especially at high concentrations. This coordinated enzymatic response highlights the complexity of insect metabolic resistance and suggests that effective control strategies may need to target multiple detoxification systems simultaneously.

## 4. Discussion

The oriental armyworm (*M*. *separata*) is a highly migratory pest that poses a severe threat to agricultural productivity and food security in Asia [31,32]. In China, it can complete multiple generations per year and rapidly evolves resistance to conventional chemical insecticides, which greatly complicates its integrated pest management [33]. Previous research has indicated that alkaloids extracted from *A. carmichaelii* are toxic to *M. separata*. Specifically, both ethanol and ether extracts of this plant exhibit strong stomach toxicity against *M. separata,* with the ether extract showing superior insecticidal activity compared to the ethanol extract [34]. Our study demonstrates that hypaconitine, a major diterpenoid alkaloid from *A. coreanum*, exerts potent and multifaceted insecticidal effects against *M*. *separata*. Beyond acute stomach toxicity and strong antifeedant activity, hypaconitine profoundly disrupts larval development, resulting in prolonged larval duration, impaired pupation. These severe morphogenetic defects suggest interference with critical endocrine or structural pathways governing metamorphosis—a phenotype not previously reported for other *Aconitum* alkaloids in *M. separata*.

Previous studies have documented insecticidal activities of *Aconitum* alkaloids against lepidopteran pests. For instance, aconitine exhibits high contact toxicity against *M. separata* [17], and alkaloids from *A. sinomontanum* achieved 70.1% antifeedant rate against this species [18]. Similarly, a derivative from *A. franchetii* showed strong antifeedant effects against *S*. *exigua* via carboxylesterase (CarE) inhibition [20]. However, none of these reports described the severe developmental abnormalities—such as malformed pupae and flightless adults—induced by hypaconitine in *M. separata*. This suggests that hypaconitine may act through distinct or additional mechanisms compared to its structural analogs.

A key finding of this work is the sustained upregulation of three major detoxification enzyme systems—CarE, glutathione S-transferases (GST), and cytochrome P450 monooxygenases (CYP450)—over 72 h following hypaconitine exposure. The absence of significant developmental delays in later instars (4th–6th), despite continuous toxin exposure, suggests that ontogenetic maturation of detoxification machinery may confer increased resilience—a phenomenon commonly observed in lepidopteran pests under xenobiotic stress. While hypaconitine exposure caused significant developmental delays in early larval instars, these effects were attenuated in later stages (4th–6th), possibly due to the ontogenetic upregulation of detoxification enzymes—a common adaptive response in lepidopteran pests under xenobiotic stress [35], prolonged activation often imposes significant metabolic costs, diverting energy and resources from growth, development, and reproduction [36,37]. Indeed, resistant insect strains frequently exhibit delayed development and reduced fitness in the absence of insecticide pressure [33]. Our observations—delayed larval development, pupal malformation, and premature adult death—are highly consistent with this “fitness cost” framework. Rather than conferring tolerance, the metabolic overdrive triggered by hypaconitine appears to exacerbate physiological stress, thereby contributing directly to its insecticidal efficacy. This mechanism is further supported by recent studies showing that diterpenoid alkaloids from *Aconitum* species directly modulate insect detoxification systems: chasmanthinine derivatives from *A. franchetii* inhibit carboxylesterase activity in *S*. *exigua* [19], while aconitine upregulates CYP450, GST, carboxylesterase, and UGT in *Nilaparvata lugens* [34], confirming that *Aconitum* alkaloids broadly interfere with xenobiotic-metabolizing enzymes.

Given that plant-derived alkaloids like those from *Aconitum* are generally biodegradable and exhibit low environmental persistence, they represent promising candidates for eco-friendly pest management [17,38]. Moreover, the combination of direct toxicity, antifeedant effects, and induction of metabolically costly detoxification responses creates a multi-layered barrier to resistance development—unlike single-site synthetic insecticides, which pests can overcome via point mutations or enzyme overexpression [39]. In the context of climate-driven pest outbreaks and expanding insecticide resistance [2,40,41], such natural compounds offer a sustainable alternative for integrated control of *M. separata* and related lepidopteran pests.

In conclusion, our findings reveal that hypaconitine’s insecticidal efficacy stems not only from its acute toxicity but also from its ability to exploit a key physiological vulnerability in pests: the fitness cost of sustained detoxification. This represents a promising paradigm for bioinsecticide design—leveraging the insect’s own defense system as a liability. Future studies should (i) employ omics approaches to elucidate the molecular targets of hypaconitine, and (ii) evaluate its environmental safety and practical efficacy in the field. Nonetheless, hypaconitine represents a promising lead compound for the development of next-generation, plant-derived insecticides in sustainable agriculture.

## Figures and Tables

**Table 1:** Probit regression analysis of the larvicidal activity of hypaconitine against second- and third-instar *M. separata* larvae.

Instar	Time (h)	Regression Equation of Toxicity	R	LC_50_ (mg/L)	95% Confidence Limits (mg/L)	χ^2^ (df = 3)	*p*-Value
2nd	48 h	y = (1.4138 ± 0.0562)x + (3.0802 ± 0.0708)	0.9969	22.7968	20.6384~25.1809	8.4787	0.0755
	72 h	y = (1.3815 ± 0.0787)x + (3.2325 ± 0.0992)	0.9936	19.0300	16.6045~21.8099	8.5522	0.0733
3rd	48 h	y = (2.5905 ± 0.2867)x + (1.3282 ± 0.4449)	0.9821	26.1475	22.0319~31.0320	6.7459	0.0805
	72 h	y = (2.4347 ± 0.3107)x + (1.6071 ± 0.4821)	0.9764	24.7491	20.2250~30.2852	6.8356	0.0773

**Table 2:** Antifeedant activity of hypaconitine against third-instar larvae of *M. separata.*

Concentration (mg/L)	24 h Antifeedant Rate (%)	48 h Antifeedant Rate (%)	72 h Antifeedant Rate (%)
5	10.40 ± 1.28(e)	13.64 ± 1.33 (f)	21.45 ± 0.38 (f)
10	25.89 ± 1.07 (d)	28.22 ± 1.29 (e)	31.34 ± 1.01 (e)
15	29.17 ± 0.42 (c)	35.56 ± 0.78 (d)	44.56 ± 1.18 (d)
20	32.78 ± 0.29 (b)	38.80 ± 0.63 (c)	49.76 ± 1.35 (c)
25	34.56 ± 0.68 (b)	43.84 ± 0.46 (b)	63.52 ± 0.23 (b)
30	39.27 ± 0.44 (a)	53.38 ± 0.89 (a)	72.99 ± 0.59 (a)

Note: Data are presented as mean ± standard error (SE). Different lowercase letters within each column indicate significant differences among treatments at *p* < 0.05 according to Bonferroni’s multiple comparison test.

**Table 3:** Probit regression analysis of the antifeedant activity of hypaconitine against third-instar *M. separata* larvae.

Exposure Time	Regression Equation	R	AFC_50_ (mg/L)	95% Confidence Limits (mg/L)	χ^2^ (df = 3)	*p*-Value
24 h	y = (1.1727 ± 0.1590)x + (3.0269 ± 0.1914)	0.9652	48.1443	33.9416~68.2901	9.3267	0.0534
48 h	y = (1.3962 ± 0.1056)x + (2.9596 ± 0.1272)	0.9888	28.9362	25.3421~33.0400	9.2142	0.0560
72 h	y = (1.7457 ± 0.2168)x + (2.8715 ± 0.2611)	0.9705	16.5683	14.2700~19.2368	9.0998	0.0587

**Table 4:** Developmental duration of *M. separata* across life stages following chronic exposure to hypaconitine.

Concentration (mg/L)	3rd Instar Duration (d)	4th Instar Duration (d)	5th Instar Duration (d)	6th Instar Duration (d)	Larval Duration (d)	Prepupal Duration (d)	Pupal Duration (d)	Adult Duration (d)
0	4.04 ± 0.06 (b)	4.44 ± 0.49 (a)	6.03 ± 0.95 (a)	6.24 ± 0.20 (a)	20.79 ± 0.69 (c)	1.81 ± 0.33 (b)	10.05 ± 0.83 (b)	11.71 ± 0.90 (a)
1	4.14 ± 0.13 (b)	4.78 ± 0.25 (a)	5.94 ± 0.34 (a)	6.28 ± 0.54 (a)	21.10 ± 0.72 (bc)	2.44 ± 0.19 (ab)	10.67 ± 0.31 (ab)	10.00 ± 0.20 (a)
2.5	4.12 ± 0.11 (b)	4.68 ± 0.39 (a)	6.00 ± 0.75 (a)	6.32 ± 0.16 (a)	21.13 ± 0.58 (bc)	2.46 ± 0.47 (ab)	10.89 ± 0.65 (ab)	10.39 ± 0.35 (a)
5	4.58 ± 0.18 (ab)	5.15 ± 0.51 (a)	5.95 ± 0.33 (a)	6.92 ± 0.25 (a)	22.60 ± 0.69 (ab)	2.65 ± 0.09 (a)	11.67 ± 0.67 (a)	10.39 ± 1.08 (a)
10	5.02 ± 0.53 (a)	5.45 ± 0.32 (a)	5.88 ± 0.23 (a)	7.39 ± 0.82 (a)	23.74 ± 0.63 (a)	2.85 ± 0.14 (a)	12.25 ± 0.55 (a)	7.92 ± 0.66 (b)

Note: Values are mean ± standard error (SE), *n* = 3 replicates. Different lowercase letters within each column indicate significant differences among treatments (*p* < 0.05, Bonferroni’s multiple comparison test). Control group: 0 mg/L hypaconitine.

**Table 5:** Effects of hypaconitine on pupation, pupal deformity, pupal weight, and adult eclosion in *M. separata.*

Concentration (mg/L)	Pupation Rate (%)	Pupal Deformity Rate (%)	Average Pupal Weight (g)	Eclosion Rate (%)
0	85.93 ± 0.07 (a)	0.00 (e)	0.4450 ± 0.02 (a)	100.00 (a)
1	64.45 ± 0.04 (c)	5.56 ± 0.10 (d)	0.4318 ± 0.02 (a)	83.33 (b)
2.5	64.88 ± 0.09 (b)	6.67 ± 0.12 (c)	0.4065 ± 0.03 (a)	79.44 ± 0.04 (c)
5	56.48 ± 0.10 (d)	7.14 ± 0.12 (b)	0.3831 ± 0.03 (a)	65.00 ± 0.09 (d)
10	55.22 ± 0.11 (e)	19.45 ± 0.05 (a)	0.3624 ± 0.04 (a)	63.89 ± 0.13 (e)

Note: Values are mean ± standard error (SE), *n* = 3 replicates. Different lowercase letters within each column indicate significant differences among treatments (*p* < 0.05, Bonferroni’s multiple comparison test). Control group: 0 mg/L hypaconitine.

## Data Availability

The data presented in this study are available within the article.
